# Randomized controlled trial demonstrates response to a probiotic intervention for metabolic syndrome that may correspond to diet

**DOI:** 10.1080/19490976.2023.2178794

**Published:** 2023-02-19

**Authors:** Hannah C. Wastyk, Dalia Perelman, Madeline Topf, Gabriela K. Fragiadakis, Jennifer L. Robinson, Justin L. Sonnenburg, Christopher D. Gardner, Erica D. Sonnenburg

**Affiliations:** a Department of Bioengineering, Stanford School of Medicine, Stanford, CA, USA; b Stanford Prevention Research Center, Department of Medicine, Stanford School of ^4^Medicine, Stanford, CA, USA; c Microbiology & Immunology, Stanford School of Medicine, Stanford, CA, USA; d Center for Human Microbiome Studies, Stanford School of Medicine, Stanford University, Stanford, CA, USA; e Chan Zuckerberg Biohub, San Francisco, CA, USA

**Keywords:** Microbiome, microbiota, probiotic, diet, metabolic syndrome, metabolomics, immune profiling

## Abstract

An individual’s immune and metabolic status is coupled to their microbiome. Probiotics offer a promising, safe route to influence host health, possibly via the microbiome. Here, we report an 18-week, randomized prospective study that explores the effects of a probiotic vs. placebo supplement on 39 adults with elevated parameters of metabolic syndrome. We performed longitudinal sampling of stool and blood to profile the human microbiome and immune system. While we did not see changes in metabolic syndrome markers in response to the probiotic across the entire cohort, there were significant improvements in triglycerides and diastolic blood pressure in a subset of probiotic arm participants. Conversely, the non-responders had increased blood glucose and insulin levels over time. The responders had a distinct microbiome profile at the end of the intervention relative to the non-responders and placebo arm. Importantly, diet was a key differentiating factor between responders and non-responders. Our results show participant-specific effects of a probiotic supplement on improving parameters of metabolic syndrome and suggest that dietary factors may enhance stability and efficacy of the supplement.

## Introduction

The incidence of several immune-related diseases, including obesity, inflammatory bowel disease (IBD), diabetes, and asthma, are rising in the US and other developed countries.^1–5^ Metabolic syndrome and prediabetes, which are often precursors to other inflammatory diseases, afflict 37% and 35% of adults in the US, respectively.^6,7^ These trends, amid exceptional gains in lifespan, suggest a deterioration of health span.^8^ The recalcitrance of metabolic disease to treatment once in advanced stages motivates identifying interventions that can reverse early signs of disease before more serious problems, such as diabetes, develop.

Researchers and clinicians have hypothesized that metabolic diseases are linked to the gut microbiome.^9–12^ Human association studies and microbiome transfer experiments in mice have demonstrated the profound effect the microbiome has on chronic inflammation and metabolism.^13–16^ The microbiome is highly responsive to a variety of dietary inputs including high-fiber, plant-based foods, meat, and non-nutritive sweeteners making it an attractive target of intervention.^17–19^ The effect of diet on the microbiome can occur rapidly, on the order of days, as well as be long lasting over years and even generations.^18,20^ Recently, a human dietary intervention demonstrated that fermented food consumption increases microbiome diversity and decreases several serum markers of inflammation, raising the possibility that probiotic-like organisms within fermented foods may benefit human health.^17^ Evidence from several randomized controlled trials further indicates that probiotics can have a beneficial effect on aspects of metabolic syndrome including blood pressure, glucose metabolism and blood lipid profiles as well as improving inflammatory biomarkers.^21^ The use of dietary intervention or supplements (e.g., traditional prebiotics and probiotics) that target the microbiome to treat diseases, such as metabolic syndrome, is attractive given their excellent safety profle.^22,23^

In this double-blinded, placebo-controlled study, we use a probiotic supplement that was specifically formulated to contain strains previously correlated with improved features of metabolic syndrome to test its ability to improve these parameters (ClinicalTrials.gov Identifier: NCT03201068).^5^ We show that the probiotic supplement did not decrease metabolic parameters as a whole, however, a subset of participants we have termed probiotic responders, had improved levels of triglycerides and diastolic blood pressure relative to the placebo group at the end of the intervention. The remainder of the intervention group participants we have termed probiotic non-responders, had increased serum glucose and insulin levels relative to the placebo group. The probiotic responders had a distinct microbiome composition and increased beta diversity compared to placebo and probiotic non-responders at the end of the intervention. Whereas non-responders’ microbiomes did not differ from the placebo groups nor were there differences in the baseline microbiome between responder groups, change in a metabolite of dopamine, homovanillic acid, was predictive of responder status. An analysis of participants’ diets revealed a difference in the consumption of certain nutrients such as sugars, lactose, and folate, all of which were consumed in greater quantities in responders relative to non-responders throughout the study. Since no nutrient was consumed in quantities that differed between responders or non-responders and the placebo group during the study, differences between responder groups were likely a result of both more consumption in the responders and less in non-responders. These data suggest that response to probiotic supplements in metabolic syndrome may be diet dependent.

## Results

### Participants with metabolic syndrome successfully completed probiotic intervention

**Figure 1.**
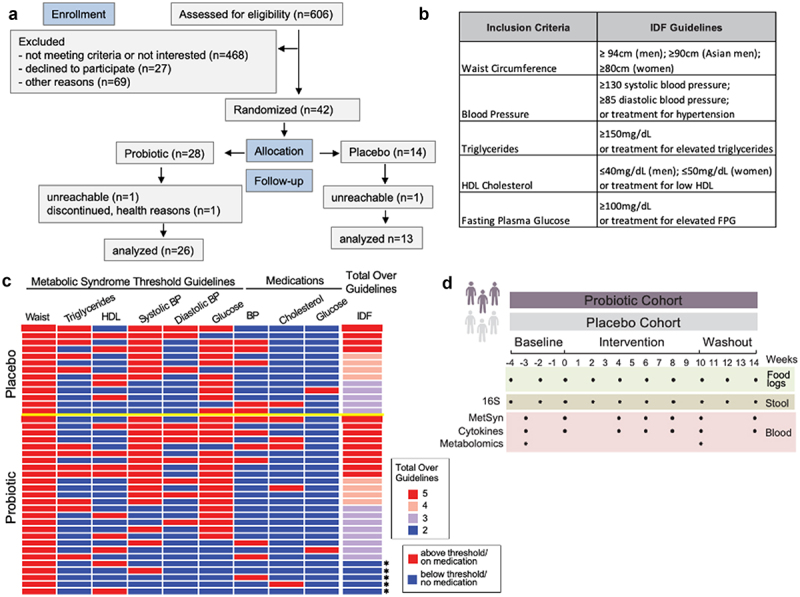
Study overview. (a) CONSORT flow diagram for enrollment, allocation, follow-up, and analysis. (b) Metabolic syndrome guidelines as defined by the International Diabetes Foundation (IDF). (c) Heat map depicting the study inclusion criteria met by participants (rows) along with medication use. Those that only satisfied 2 criteria are indicated by an asterisk. Yellow line separates placebo from probiotic arm. HDL = high density lipoprotein; BP = blood pressure (d) Stool and blood samples along with participant food logs were collected over an 18-week period for microbiome and immune system measurements.

To determine the effect of a probiotic supplement on adults with elevated parameters of metabolic syndrome, as defined by the International Diabetes Federation (IDF) Guidelines,^24^ we recruited participants for an 18-week randomized, double-blind, placebo-controlled study. Of over 600 individuals initially expressing interest, 42 maintained interest after learning about all the study requirements, met all inclusion and exclusion criteria, and agreed to be enrolled; 39 completed all the study procedures (Figure 1a and Figure 1b).^5^ Participants were required to have central obesity (as defined by the International Diabetes Foundation), in addition to at least one of the following: elevated blood pressure, elevated fasting blood sugar, low HDL cholesterol, elevated triglycerides, or be prescribed medication to control these conditions and were randomized into the probiotic arm (n = 26) or placebo arm (n = 13) (Figure 1c, Table S1). Note that metabolic syndrome, as defined by the IDF, requires three or more parameters above the specified threshold. The primary outcome of the study was a change in metabolic syndrome parameters in participants with metabolic syndrome from the beginning (week 0) to the end (week 10) of intervention. Since five participants (all randomized to the probiotic treatment arm) had only two parameters above the IDF guidelines, they were excluded from analysis for primary outcome since they did not meet the three parameters above the IDF guidelines definition for metabolic syndrome. All participants provided blood and stool samples during the four weeks prior to the intervention (baseline), the 10-week intervention period in which participants took a probiotic supplement or placebo once daily, and the 4-week washout period in which no probiotic or placebo was taken (Figure 1d). Both arms had high adherence with 90% of the placebo arm and 88% of probiotic arm taking at least 80% of the supplement or placebo. The probiotic supplement contained a proprietary blend of three probiotic strains (*Limosilactobacillus reuteri NCIMB 30242, Lactiplantibacillus plantarum* UALp-05™, and *Bifidobacterium animalis* subsp. *lactis* B420™), which were verified through sequencing (Fig. S1A), and at least 20 billion colony forming units per capsule. These strains were chosen due to reports of beneficial effects on features of metabolic syndrome including improved glucose metabolism, weight loss, and improved blood lipid profiles.^25–30^

Participants were instructed to keep their exercise and diet unchanged during the study, logged three days of dietary intake every two weeks (10 entries total), were weighed at all seven clinic visits, answered questionnaires to assess quality of life and gastrointestinal symptoms, and took a visual-cognitive test. Data were collected every 2–4 weeks during the seven clinic visits and through 10 at-home data collection points. Participants maintained stable nutritional intake, weight, and total caloric intake across the entire study (Table S2, Fig. S1B, C). As measured by the Gastrointestinal Symptom Rating Scale (GSRS) assessment, participants in the probiotic arm reported a slight increase in appearance of loose stools relative to the placebo arm during the intervention (1.5 ± 0.5 vs. 1.2 ± 0.4 for probiotic and placebo groups, respectively; *p* = 0.04, unpaired *t*-test; 1 = normal; 2 = somewhat loose; 3 = runny; 4 = watery).^31^ Most participants in the probiotic arm (71%) reported loose, runny, or watery stool at least once during the intervention versus 43% of participants in the placebo arm. There were no differences between arms in the quality of life or visual-cognitive assessment from baseline to end of intervention.

### The probiotic did not have arm-wide effects in metabolic syndrome or immune parameters but divided the probiotic arm into responders and non-responders

**Figure 2.**
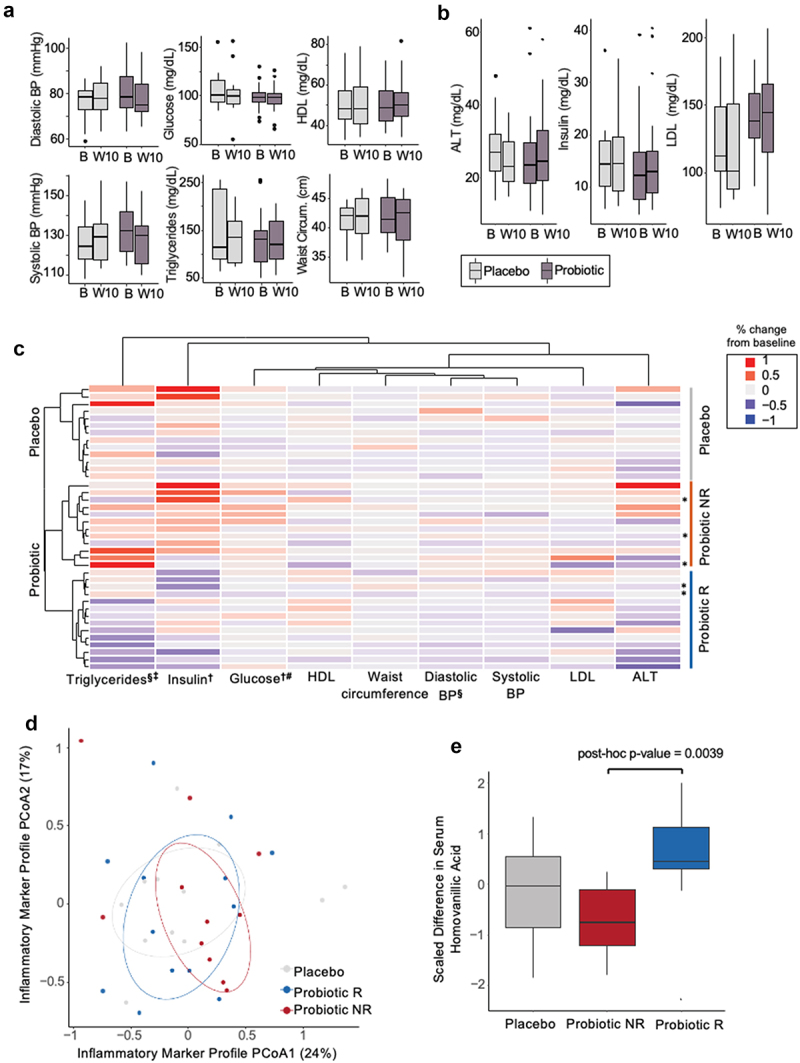
MetSyn parameters did not improve in the probiotic arm, but participants cluster into responder and non-responder groups. (A) MetSyn parameters and (B) alanine aminotransferase (ALT), insulin, and LDL cholesterol at baseline (“B”, week 0) and end of intervention (“W10”, week 10) for probiotic (n=21; participants that had at least 3 elevated parameters and thus met the definition of metabolic syndrome) and placebo arms (n=13). (C) Change from baseline average (weeks -4, -2, 0) to end of intervention (weeks 8, 10) for each participant in placebo, probiotic responder (R), or probiotic non-responder (NR) groups. All probiotic participants (n=26) plotted including those with only 2 elevated parameters (‪ indicates participants that only satisfied 2 criteria for MetSyn). Each row is a participant and is hierarchically clustered by parameters shown. § indicates significant difference between average baseline and end of intervention within the probiotic responders (paired t-test < 0.05). ‡ indicates significant difference between average percent fold change between probiotic responders and placebo (unpaired t-test < 0.05). † indicates significant difference between average baseline and end of intervention within the probiotic non-responders (paired t-test < 0.05). # indicates significant difference between average percent fold change between probiotic non-responders and placebo (unpaired t-test <0.05). (D) Principal coordinate analysis of Euclidean distances for % change in inflammatory marker from baseline (week -3) to end of intervention (week 10) for all participants. Circles denote 50% confidence level for a multivariate t-distribution. (E) Change in serum homovanillic acid levels from baseline (week -3) to end of intervention (week 10) for placebo, probiotic non-responders (NR), and responders (R) (unpaired t-test, post-hoc p-value = 0.004).

The primary outcome of change in metabolic syndrome parameters in participants with metabolic syndrome (including only the 21 participants from the probiotic arm that had at least three parameters above the IDF guidelines and all 13 placebo arm participants) from beginning (week 0) to end (week 10) of intervention was not significant in the probiotic arm (*n* = 21, Figure 2a, Table S3, S4).^5^ There were no significant changes in diastolic blood pressure, systolic blood pressure, fasting glucose levels, triglycerides, HDL cholesterol, or waist circumference over time within the probiotic arm (*n* = 21) between baseline and intervention (paired *t*-test) or between arms (unpaired *t*-test) at the end of intervention. Blood alanine transaminase (ALT), insulin, and LDL cholesterol, elevated levels of which are associated with deteriorating health and type 2 diabetes,^32–34^ also did not change between week 0 and week 10 within the probiotic arm (*n* = 21), nor between arms (paired and unpaired *t*-test, respectively) (Figure 2b). When including the five participants with only two markers of metabolic syndrome to the analysis (*n* = 26, probiotic arm), the lack of significance between the probiotic and placebo arms remained (data not shown).

We next leveraged aspects of our study design (e.g., the longitudinal sampling, dietary records, ability to perform microbiome profiling) in a discovery analysis process to reveal trends that could inform possible probiotic efficacy and hypotheses for future studies.^5^ For all analyses presented from this point forward, we utilized all participants’ data (*n* = 13, placebo arm; *n* = 26 probiotic arm) including the five participants with only two elevated metabolic syndrome parameters, since these analyses are discovery based and were not part of the original primary outcome. To reduce intra-participant variability, we averaged timepoints at each phase (average baseline = weeks −4, −2, 0; average end of intervention = weeks 8, 10) to compare metabolic syndrome parameters (Figure 2c). We performed hierarchical clustering of each participant’s change in these nine metabolic syndrome parameters from their average baseline to their average end of intervention levels. This analysis reveals a clear separation between the probiotic and placebo arms and two subsets of participants in the probiotic arm. In one group of probiotic arm participants (*n* = 14), we observed within participant decrease in triglycerides and diastolic blood pressure over time (paired *t*-test, *p*-value adj. = 0.04 and 0.04, respectively; Table S5). We termed this subset of participants responders. The other subset of probiotic participants (*n* = 12), which we termed non-responders, did not have within participant changes in triglyceride or diastolic blood pressure over time (paired *t*-test, *p*-value adj. = 0.28 and 0.91, respectively). However, the non-responders did have a significant increase in insulin and glucose levels over time (paired *t*-test, *p*-value adj. = 0.03 and 0.03, respectively). There were no changes within the placebo arm in any of the metabolic syndrome parameters we measured (Table S5), nor were there differences in demographic, anthropometric data, or medication use between responders and non-responders at study enrollment (Table S6, Table S7). Furthermore, the five participants with only two elevated metabolic syndrome parameters were divided equivalently between the responder groups (Figure 2c).

Unlike the probiotic arm, the placebo arm did not separate into clear groupings, with the 13 participants most naturally splitting into four groups one of which only contained a single participant (Figure 2c). For this reason, we compared the responder and non-responder groups to the natural variation in the entire placebo group. Comparisons between the responder group and the placebo arm, reveals declining triglycerides in the responder group over time relative to the placebo group (unpaired *t*-test, *p*-value adj. = 0.02; Table S8), whereas changes in triglycerides are not different between the non-responder and placebo groups (unpaired *t*-test, *p*-value adj. = 0.8). Notably, the change in triglycerides is negatively correlated to baseline triglyceride levels in the responder group only (Pearson correlation, *p*-value = 0.03, corr = −0.53), suggesting that higher levels of triglycerides at baseline corresponded to a larger decrease during the intervention (Fig. S2A). This association is specific to the responders as it is not seen for non-responders, the entire probiotic arm, or the placebo arm. Triglyceride levels at baseline are not different between the responders and non-responders (unpaired *t*-test, *p*-value = 0.3). While responders had declining diastolic blood pressure over time, this decline is not significant when compared to changes in diastolic blood pressure in the placebo group (unpaired *t*-test, *p*-value = 0.27; Table S8). Unlike changes in triglycerides, the correlation between baseline values of diastolic blood pressure and percent change is also not significant in diastolic blood pressure. Comparison of the non-responder group to the placebo group reveals increased serum glucose over time indicating that the non-responders fared worse than the placebo in this metric (unpaired *t-*test, *p*-value adj = 0.045; Table S5). Insulin levels were not significantly different between non-responders and the placebo group despite increasing over time in the non-responder group (unpaired *t*-test, *p*-value = 0.68). In assessing the Homeostatic Model Assessment for Insulin Resistance (HOMA-IR), which takes into account fasting insulin and glucose levels and is a marker for health outcomes related to metabolic syndrome, we found no difference in this metric at the end of the intervention between the responder or non-responder group and placebo (unpaired *t*-test, *p*-value = 0.16 and 0.58, respectively).

In summary, the probiotic treatment did not reduce parameters of metabolic syndrome as a whole. However, a subset of participants, the probiotic responders, did exhibit decreases in triglycerides and diastolic blood pressure over the course of the intervention with the decrease in triglycerides significant relative to the placebo group. The probiotic non-responders increased blood glucose and insulin levels over time, with the increase in glucose significant relative to the placebo group. These data are consistent with some participants improving with probiotic supplementation and others faring worse relative to no probiotic intervention.

While there were not cohort-wide improvements in metabolic syndrome parameters in the probiotic arm relative to placebo, we wondered whether there were observable changes in aspects of participants’ immune system in response to the probiotic. Increased inflammation, as determined by elevated levels of several circulating pro-inflammatory cytokines and chemokines, has been linked to metabolic syndrome.^35^ Therefore, we reasoned that changes in markers of inflammation could be an indication that the probiotic supplement was affecting participants and could be preceding a beneficial effect to a greater proportion of participants given a longer intervention period. Participants’ serum was analyzed for 92 different circulating cytokines, chemokines, and additional immune modulators (Table S9).^5^ No changes in immune features from baseline to end of intervention within participants were observed in either the probiotic or placebo arm, nor in the responder or non-responder subgroups. Direct comparison of placebo and probiotic arms at the end of the intervention also did not reveal any differences (data not shown). Similarly, participants’ serum immune markers did not cluster based on treatment arm (adonis, *p*-value = 0.7) or probiotic response group (adonis, *p*-value = 0.6) indicating that the probiotic intervention did not decrease inflammation in the responder group nor increase inflammation in the non-responders (Figure 2d).

We next wondered whether the probiotic affected circulating metabolites, many of which serve as markers of physiological and metabolic processes in humans. We ran untargeted metabolomics using our recently reported high-throughput liquid chromatography-mass spectrometry (LC-MS) approach^36^ on serum samples and compared metabolic profiles at baseline and end of intervention. This comparison reveals distinct clustering between the placebo arm, probiotic responders, and probiotic non-responders (adonis, *p*-value = 0.046, Fig. S2B). A random forest recursive feature-elimination algorithm further reveals that change a single metabolite, homovanillic acid (HVA), a metabolite of dopamine, can predict probiotic responder or non-responder with 71% accuracy. The random forest model predicted the probiotic versus placebo treatment arms with only 65% accuracy using 100 different metabolites. Changes in serum HVA differed between responders and non-responders (post-hoc, unpaired *t*-test *p*-value = 0.004; Figure 2e), but not between responders or non-responders and placebo (post-hoc *p*-value = 0.13, 0.18; respectively). This difference in HVA was driven by decreasing HVA over time within the non-responder group (paired *p*-value = 0.01), while remaining unchanged in the placebo and responder groups (paired *p*-value = 0.53, 0.14; respectively).

### Microbiome profiles shift in the probiotic responders

**Figure 3.**
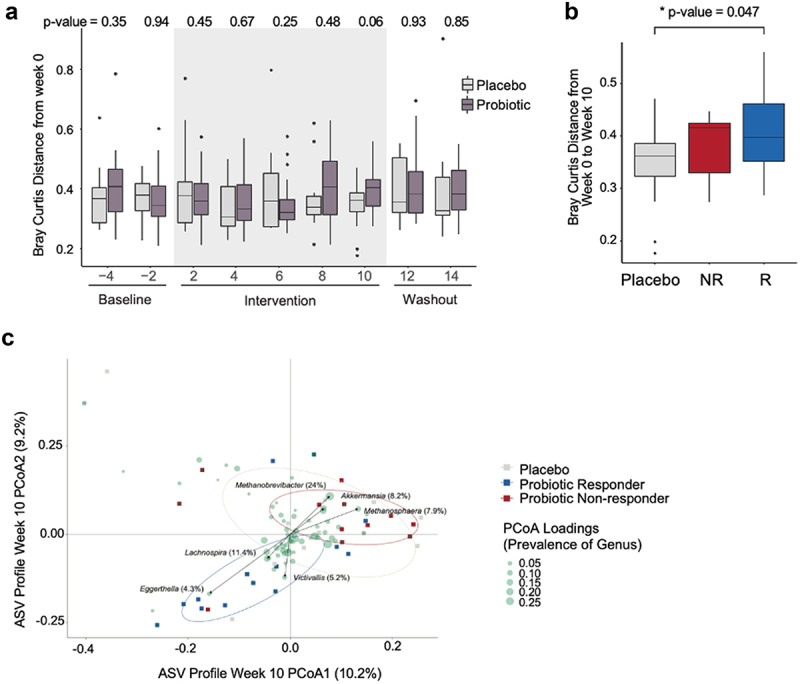
Probiotic responders exhibit distinct microbiome profiles. (a) Within-participant Bray-Curtis distance of amplicon sequenced variants (ASVs) relative to the first baseline time point (week 0) plotted over time by arm (unpaired t-test p values for each time point between arms are shown) (n=13 placebo; n=26 probiotic). (b) Within-participant Bray-Curtis distance, week 0 vs. week 10 by arm and response group (n=14 R; n=12 NR). (c) Bray-Curtis distance of the ASVs for the placebo, probiotic responders and non-responders at the end of the intervention (week 10) are plotted. Circles denote 50% confidence level for a multivariate t-distribution. ASVs driving the separation between groups are depicted as green circles, collapsed to average loading across genus. Size of green circles indicate prevalence (% present across all samples at week 10).

To characterize participants’ microbiomes, fecal samples were subjected to 16S rRNA amplicon sequencing and amplicon sequenced variants (ASVs) were assigned.^5^ There were no differences in microbiota alpha diversity between study arms at all phases of the study or from baseline to end of intervention within participants (Fig. S2C). To determine whether participants’ microbiota composition changed over the course of the study, we calculated the Bray Curtis distance from each participant’s microbiota at week 0 to all other time points and found no significant changes in either arm (Figure 3a). Similarly, when compared between arms, there was no difference in Bray Curtis distance from week 0 to any other time point (Figure 3a). At the end of the intervention, there was a trend toward significance between the arms (unpaired *t*-test; *p*-value = 0.06) indicating that the microbiota of those in the probiotic arm may have begun to change from their baseline composition relative to the placebo group. Given that we had identified metabolic syndrome responders in the probiotic group, we wondered whether response was linked to the microbiota. There was no difference in the baseline microbiota composition between responders and non-responders (Bray–Curtis distance, unpaired *t*-test *p*-value = 0.15). However, we observed an increase in Bray–Curtis distance from baseline to the end of intervention in the responders relative to placebo (unpaired *t*-test, *p*-value = 0.047), with non-responders exhibiting a non-significant but intermediate level of microbiota change (Figure 3b). These data suggest that the microbiota of the responders in the probiotic arm shifted from baseline to the end of the intervention. At the end of the intervention, the microbiota composition of the responders differed from the placebo or non-responders (adonis, *p*-value = 0.05); whereas the microbiota of the non-responders and placebo groups did not differ (adonis, *p*-value = 0.40) (Figure 3c). Many of the ASVs differentiating responders from non-responders and placebo groups, were of low prevalence (found in 25% or less participants), however, some have been previously associated with health (Table S10). Taxa driving the responder group, *Eggerthella*, is associated with positive effects on inflammation and obesity in humans,^37^ and *Lachnospira*, along with *Eggerthella*, are associated with better outcomes in patients with inflammatory bowel disease receiving anti-TNF therapy.^38^ *Akkermansia*, a driver of the non-responder group, is associated with increased levels of inflammation, although also associated with protection from obesity.^39,40^ Other non-responder drivers, *Methanosphaera* and *Methanobrevibacter* are more abundant in patients with inflammatory bowel disease.^41^

Despite response group-specific changes to the microbiota, neither baseline microbiota composition (ASV relative abundance) nor alpha diversity could predict response to the probiotic (leave-one-out cross-validated random forest). Similarly, baseline measurements of blood pressure, waist circumference, serum metabolomics, and circulating inflammatory and gender were not correlated with response group (SAM, FDR > 0.05).

### Dietary intake separates probiotic responders vs. non-responders

**Figure 4.**
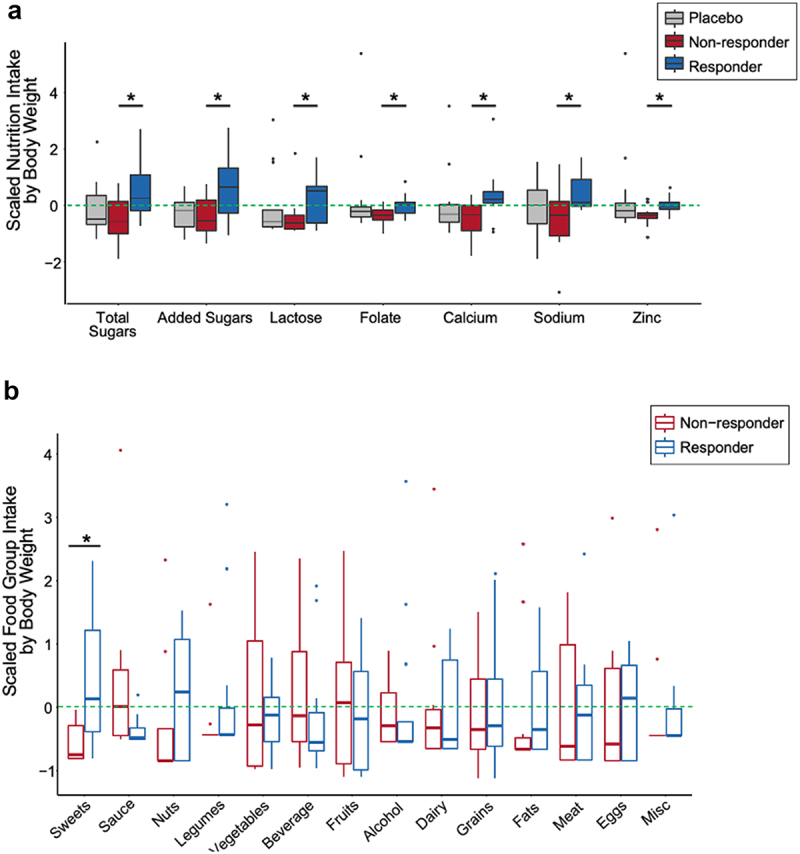
Dietary intake differentiating probiotic responders and non-responders. (A) Nutrients that differ between probiotic responders (n=14) and non-responders (n=12) across the entire study (weeks -4 through 14), normalized by median body weight (kg) (siggenes, FDR < 0.05, q-value < 0.1). Scaled nutrient intake is the mean intake value subtracted from each value and divided by the standard deviation. The line within the box plot represents the median value. (B) Scaled average food group intake (weeks 0 and 10) in which the mean value is subtracted from each value and divided by the standard deviation normalized for median body weight between responders and non-responders (sweets; unpaired t-test, p-value = 0.010). The line within the box plot represents the median value.

While participants did not change their diet over the course of the study (linear mixed effects modeling, *p*-value adj. > 0.05; Table S2), we wondered whether aspects of participants’ diet corresponded to probiotic response.^5^ There was no difference in median intake for 108 dietary parameters across all time points between arms, nor between probiotic responders, non-responders, and the placebo group. Furthermore, there was no difference in probiotic supplement compliance between responder groups with 94% and 91% probiotic capsules consumed for responder vs. non-responders, respectively (*p*-value = 0.5). We used the 16S rRNA sequences to detect probiotic strains in stool (i.e., only detectable in the intervention group during the intervention) and saw no difference in the presence or absence of the probiotic strains assigned ASVs within the microbiome of responder and non-responders (chi-square *p*-value = 0.4). However, when dietary parameters were normalized by weight for each participant, 14 nutrients, all of which were higher in the responders relative to non-responders, differentiated the two groups (Table S11; SAM, FDR ≤ 0.05, q-value ≤ 0.1). These 14 nutrients did not differ between either responder group and the placebo group indicating that differences were likely a result of slightly increased consumption by the responders and slightly decreased consumption by non-responders (Table S11). The 14 nutrients were subsetted to seven non-redundant nutrients shown in Figure 4a, as determined by a nutritionist (Figure 4a; Fig S3). To identify food sources of the seven nutrients that differentiated response group, we averaged total food intake at week 0 and week 10 and assigned these foods to 14 different food groups, such as dairy, fruits, meat, etc. (Table S12). Of these 14 food groups, only intake of sweets differed between the probiotic responders and non-responders with increased sweets consumption in the responders relative to non-responders (Figure 4b; unpaired *t*-test, *p*-value = 0.01). These data are consistent with the nutrient data indicating differences in sugar consumption between responders non-responders.

Interestingly, despite lower sugar consumption in the non-responders relative to responders, the non-responder group had elevated blood glucose over the course of the study and relative to the placebo group. Conversely, probiotic responders consumed more sugar than non-responders, yet their blood glucose was not different from the placebo group. These differences highlight an interesting combination of increased blood sugar despite lower sugar consumption in the non-responders.

## Discussion

Probiotics supplements are growing in popularity globally^6^ despite conflicting clinical data as to their efficacy for a number of health conditions.^42,43^ Benefit from probiotic supplementation has been strongest for gut-specific diseases such as diarrhea, irritable bowel syndrome, and *Clostridioides difficile* infection.^44–47^ The ability of probiotics to impact metabolic syndrome in human subjects has shown only modest benefits in clinical characteristics and inflammatory markers.^21,48–50^ Here, we describe a double-blinded, placebo controlled human study examining the effects of a probiotic supplement designed specifically to treat metabolic syndrome, combining high dimensional, longitudinal microbiome and immune profiling. We did not observe a probiotic arm-wide improvement in metabolic syndrome parameters nor a decrease in circulating markers of inflammation in the 21 participants with 3–5 elevated metabolic syndrome parameters nor within the expanded group of 26 participants including the five with only two elevated metabolic syndrome parameters. However, we identified a subset within the probiotic arm with lowered triglycerides and diastolic blood pressure, a group we termed probiotic responders. Interestingly, the remaining participants in the probiotic arm, the probiotic non-responders, had higher levels of serum glucose and insulin at the end of the intervention, indicating that for these participants the probiotic could be having a detrimental effect. While some studies have described a potential benefit in glycemic control with probiotic supplementation, there does not appear to be a consensus among studies,^51^ which would be consistent with a portion of the population being responsive to probiotics and others being non-responsive or adversely-responsive. In general, probiotic supplements are considered extremely safe, however, there have been reports that probiotics can lead to acute issues such as sepsis and gastrointestinal issues in individuals with compromised immune systems.^22^ Similarly, probiotic supplementation can hinder microbiome recovery after antibiotic treatment.^52^ Whether probiotic supplementation could be detrimental in some individuals with metabolic syndrome remains unknown, but as the field of personalized medicine and nutrition advances, understanding how an individual’s underlying physiology impacts probiotic efficacy will likely be important in treating conditions like metabolic syndrome.^53,54^

In searching for markers that distinguish response groups, we found that circulating levels of homovanillic acid (HVA) differentiated responders and non-responders with fairly high accuracy (71%). This difference in HVA between groups appears to be due to declining levels of HVA over the course of the study in the non-responder group. HVA is a product of human metabolism, a major metabolite of dopamine, and its circulating levels are indicative of dopaminergic activity.^55^ Declining levels of HVA could be an indication of decreased levels of dopamine. This outcome is counter to a clinical trial in autistic children in which HVA levels increased with a probiotic plus prebiotic (fructo-oligosaccharide) intervention, however this group of children had lower HVA relative to healthy controls.^56^ It is unknown whether metabolic syndrome is coupled to lower HVA relative to healthy controls or whether coupling a prebiotic to the probiotic intervention here would have impacted HVA levels. Interestingly, HVA can also be produced by the microbiota from dietary polyphenols (e.g., from grapes) raising the possibility that differences in HVA levels between responders and non-responders could be driven by differences in diet and/or microbiome metabolic activity between the two groups.^57^

The microbiome is linked to host metabolism, and dietary interventions that target the microbiome can alter human immune status and metabolism.^17,58–63^ While diet directly influences microbiome composition and diversity, probiotic supplements, do not appear to have as profound an effect on the microbiome.^64,65^ Similarly, we find that probiotic consumption did not alter participants’ microbiota composition or diversity as a whole. This finding contrasts with increased microbiota diversity observed in individuals consuming fermented foods, containing bacteria that are similar phylogenetically to many probiotics.^17^ However, increased diversity observed during the fermented food intervention was not from ingested fermented food-associated bacteria indicating that new species were either acquired or increased to detectable abundance upon fermented food consumption. Unlike non-responders or participants in the placebo group, the microbiota of probiotic responders did shift in composition from their baseline state to the end of the intervention. In fact, by the end of the intervention the microbiomes of responders were different than those in the placebo and non-responder groups despite there being no difference in the baseline microbiome between groups. Interestingly, one taxon that drove microbiome differences between responders and non-responders was *Akkermansia*, which was enriched in the non-responders relative to responders. There have been several studies linking *Akkermansia muciniphila* with improved metabolic health (reviewed in^66^). However, *A. muciniphila* has also been associated with poor glycemic control, colitis, and pathogen induced inflammation among other negative outcomes (reviewed in^67^). Further investigation is required to determine whether differences in *Akkermansia* or other taxa between responders and non-responders could be mediating the disparate effect of the probiotic between these two groups.

One predictive feature of probiotic response group was differences in dietary intake of total and added sugars, lactose, and sucrose throughout the study. Probiotic responders consumed more of these nutrients relative to non-responders, however it is important to note that since consumption of these nutrients did not differ between either group and the placebo group, differences were likely driven by both increased consumption by responders and decreased consumption by non-responders. This difference in sugar consumption between groups is counter-intuitive given the link between sugar consumption and poor metabolic health.^68^ In other words, we might have expected that participants consuming less sugar, the non-responder group, would have lower serum glucose and insulin over time relative to responders, which was the opposite of what we observed. Carbohydrates can enhance growth and survival of a probiotic strain of *Lactococcus lactis*.^69^ Specifically, sucrose and lactose promoted growth and survival of this strain using *in vitro* conditions that mimicked the gut. These reports coupled to our data raise the possibility that, in this study, increased consumption of sugars enhanced probiotic survival and growth within the gut of the responders relative to non-responders. In other words, consumed sugars may have functioned as a prebiotic that in combination with the probiotic supplement could have produced a synbiotic effect.^70^ Most prebiotics are complex carbohydrates that are recalcitrant to degradation and absorption within the small intestine, making them available for fermentation by colonic microbes. Simple sugars, such as those consumed more by the responder group, are more easily absorbed by the host within the small intestine and thus less available to microbes within the colon. However, small intestinal fermentation of simple carbohydrates by probiotic species is possible given reports of administered probiotics strains residing within the small intestine of humans for several days.^71^ Therefore, it is possible that small intestinal residing probiotic strains fermented ingested sugars both decreasing the amount of absorbable sugar and potentially producing fermentation end products such as short-chain fatty acids that can impact human health.^72^ We did not detect differences in the presence of probiotic strains in fecal samples between response groups, however, we do not know if or how the probiotic may have influenced the small intestinal microbiome.

The lack of an arm-wide response to the probiotic intervention could be a result of participant heterogeneity, insufficient sample size, or insufficient length of probiotic intervention. Longer probiotic interventions (> 3 months) have been shown to be beneficial in controlling fat mass and lipid profiles.^30^ Participant heterogeneity related to diet, microbiome composition, and other physiological differences could be a factor in why some participants’ metabolic syndrome parameters improved with probiotic intervention and other participants appeared to worsen. Understanding the baseline characteristics that are predictive of a probiotic responder versus a non-responder, like those identified to predict postprandial glycemic response, would be valuable in maximizing probiotic efficacy.^73^ We did not identify any baseline microbiome, immune, or metabolic characteristics associated with responder status, other than differences in sugar consumption. However, the multi-omic nature of this study allowed us to identify a circulating metabolite, homovanillic acid, in addition to sugar consumption as possible mediators of probiotic response in individuals with metabolic syndrome.

## Materials and methods

### Recruitment and selection of participants

Participant recruitment was conducted in the local community via online advertisement and radio in different community groups, as well as e-mails to past research participants that consented to being contacted for future research. The current study assessed 606 participants for eligibility. They completed an online screening questionnaire and a clinic visit between September 2017 and June 2018. The primary inclusion criteria included the following: age ≥ 18 years.

At least two of the five criteria used to diagnose metabolic syndrome as defined by either the ATP-III Guideline, or by the International Diabetes Federation Guideline:

1. Increased waist circumference, with ethnic-specific waist circumference cut-points:

White and all other ethnic groups – men ≥ 94cm; women ≥ 80 cm

South Asians, Chinese, and Japanese – men ≥ 90cm; women ≥ 80 cm

2. Triglycerides ≥150 mg/dL (1.7 mmol/L) or treatment for elevated triglycerides

3. HDL cholesterol <40 mg/dL (1.03 mmol/L) in men or <50 mg/dL (1.29 mmol/L) in women, or treatment for low HDL

4. Systolic blood pressure ≥130, diastolic blood pressure ≥85, or treatment for hypertension

5. FPG ≥100 mg/dL (5.6 mmol/L) or previously diagnosed type 2 diabetes; an oral glucose tolerance test is recommended for patients with an elevated fasting plasma glucose, but not required

Otherwise, healthy subjects willing and able to provide blood and stool specimens, able to provide signed and dated informed consent, and be willing to follow protocol.

Exclusion criteria included the following:

· Body mass index ≥ 40

· Vital signs outside of acceptable range at screening visit, i.e., blood pressure >159/99, oral temperature ≥100°F, pulse >100, LDL >190 mg/dL.

· Use of any of the following drugs within the last 6 months: systemic antibiotics (must be discontinued and avoided for 2 months prior to the study start), antifungals, antivirals or antiparasitics (intravenous, intramuscular, or oral); oral, intravenous, intramuscular, nasal or inhaled corticosteroids; cytokines; methotrexate or immunosuppressive cytotoxic agents; large doses of commercial probiotics consumed (greater than or equal to 108 cfu or organisms per day) – includes tablets, capsules, lozenges, chewing gum or powders in which probiotic is a primary component. (Must be discontinued and avoided for one month prior to the study start.) Ordinary dietary components such as fermented beverages/milks, yogurts, foods do not apply.

· Acute disease at the time of enrollment (defer sampling until subject recovers). Acute disease is defined as the presence of a moderate or severe illness with or without fever. Chronic, clinically significant (unresolved, requiring ongoing medical management or medication) pulmonary, cardiovascular, gastrointestinal, hepatic or renal functional abnormality, as determined by medical history, including Type I diabetes. (Type II diabetes ok if controlled by metformin or diet).

· History of cancer except for squamous or basal cell carcinomas of the skin that have been medically managed by local excision (allowed if cancer was several years past and not requiring in continual care).

· Unstable dietary history as defined by major changes in diet during the previous month, where the subject has eliminated or significantly increased a major food group in the diet. Recent history of chronic alcohol consumption defined as more than five 1.5-ounce servings of 80 proof distilled spirits, five 12-ounce servings of beer or five 5-ounce servings of wine per day.

· Positive test for HIV, HBV or HCV.

· Any confirmed or suspected condition/state of immunosuppression or immunodeficiency (primary or acquired) including HIV infection.

· Major surgery of the GI tract, with the exception of appendectomy, in the past five years.

· Any major bowel resection at any time. History of active uncontrolled gastrointestinal disorders or diseases including: inflammatory bowel disease (IBD) including ulcerative colitis (mild-moderate-severe), Crohn’s disease (mild-moderate-severe), or indeterminate colitis; irritable bowel syndrome (IBS) (moderate-severe); persistent, infectious gastroenteritis, colitis or gastritis, persistent or chronic diarrhea of unknown etiology, *Clostridioides difficile* infection (recurrent) or *Helicobacter pylori* infection (untreated); chronic constipation.

· Female who is pregnant or lactating.

· History of gallbladder removal

All study participants provided written informed consent. The study was approved annually by the Stanford University Human Subjects Committee. Trial was registered at ClinicalTrials.gov, identifier: NCT03201068.

### Randomization, blinding, food logs, and surveys

Sample size was determined by budget, and we utilized a 2:1 (probiotic:placebo) ratio for randomization in order to increase the number of participants in the active arm to perform within arm comparisons over time. Both the participants and the study team administering the supplements were blinded. Participants were randomized into probiotic or placebo arms using a random number generator in Excel by a statistician from the Quantitative Science Unit at Stanford School of Medicine not involved in the intervention or data collection, the randomization assignments were stratified by sex in blocks of 3, there were no additional stratifications performed. Once participants completed the baseline data collection, they were assigned their arm based on the randomization described above and provided with bottles containing either probiotic or placebo and labeled with only bottle number, participant number, and appointment date. The bottle and labels were generated by the probiotic manufacturer. Participants were unblinded by e-mail after completion of the entire protocol. Investigators were unblinded after primary outcome calculation. Participants were asked to keep detailed food logs 3 days per week (two weekdays and one weekend) every other week through the duration of the study using the HIPAA compliant HealthWatch360 app (https://healthwatch360.gbhealthwatch.com/), which has a research portal designed to collect dietary data and allowed the dietitian to interact with study participants and view, download, and analyze data collected with the app. A trained dietitian reviewed the entries with participants to assess accuracy of entries and portions. An average of the 3 days was used for each time point. The dietician transferred the diet data that was inputted by the participant in HealthWatch360 into the USDA National Nutrient Database for Standard Reference (NDSR), which includes nutrients (macro and micro) in accurate amounts, in order to generate nutrient data used for the study. Nutrient data from HealthWatch 360 was not used in the analysis since it lacks nutrient data for some food entries. NDSR appendix 10 was used to classify foods into food groups. Broad annotation of ~100 food groups into categories of sweets, sauce, nuts, legumes, vegetables, beverages, fruits, alcohol, dairy, grains, fats, meat, eggs, and miscellaneous was done by hand by the data analysis team. Participants filled out gastrointestinal symptoms surveys (GSRS)^31^ and symptom changes^74^ every 2 weeks. The following validated health surveys were used by participants: PROMIS v1.1 global health, PROMIS v1.0 – fatigue, WHO well-being index, PROMIS applied cognition short form, Perceived Stress Scale,^75^ and the International Physical Activity Questionnaire.^76^

### Probiotic formulation and instructions

The probiotic supplement used was a proprietary blend of three probiotic strains, specifically formulated based on a literature review of previous studies in which a beneficial effect was seen with a particular strain and metabolic syndrome. *Limosilactobacillus reuteri NCIMB 30242*, LRC™ (6 billion CFUs/dose) was chosen due to documented clinical evidence in lowering LDL-cholesterol, reducing sterol absorption, affecting the inflammation cascade (anti-inflammatory), promoting digestive health scores, and enhancing circulating vitamin D levels. *Lactiplantibacillus plantarum* UALp-05™ (4 billion CFUs/dose) was chosen due to the wide-ranging evidence in irritable bowel syndrome subjects and providing immune benefits. *Bifidobacterium animalis* subsp. *lactis* B420™ (10 billion CFUs/dose) was chosen due to evidence for clinical outcomes including improved glucose metabolism, weight loss, reduced trunk fat, reduction in endotoxemia induced tissue inflammation. The excipient was microcrystalline cellulose. The placebo did not include the probiotic strains but included additional microcrystalline cellulose to replace the probiotic culture powder and was otherwise identical to the test capsules. Participants were instructed to take the supplement once a day, every day for the 10 weeks of intervention, while keeping their diet, weight, and exercise levels constant. They were asked to consume the supplement just before the meal that is typically their largest meal of the day. If participants missed their regular dose, they were instructed to take the capsule as soon as they remembered before the end of the day, preferably with a meal. If participants missed a dose entirely for the day, they were instructed to skip the dose and continue with their regular dose schedule on the following day.

### Specimen collection

Stool samples were collected every two weeks from week −4 through week 14. All stool samples were kept in participants’ home freezers (−20°C) wrapped in ice packs, until they were transferred on ice to the research laboratory and stored at −80°C. Blood samples were collected at seven time points: weeks −3, 0, 4, 6, 8, 10, and 14. Blood for serum was collected into an SST-tiger top tube, spun at 1,200xg for 10 minutes, aliquoted, and stored at −80°C. Blood for plasma was collected into an EDTA tube, spun at 1,200×*g* for 10 min, aliquoted, and stored at −80°C.

### 16S amplicon sequencing

DNA was extracted from stool using the MoBio PowerSoil kit according to the Earth Microbiome Project’s protocol^77,78^ and amplified at the V4 region of the 16S ribosomal RNA (rRNA) subunit gene and 240 nucleotides (nt) Illumina sequencing reads were generated. Samples with less than 1,000 reads (4 samples out of 396 removed) were filtered out, leaving an average of 17,182 reads per sample. There was an average of 13,080 reads per sample recovered after filtering, denoising, removing chimeras, and merging paired reads. Sequencing data were demultiplexed using the QIIME pipeline version 1.8^78^ and amplicon sequence variants (ASVs) were identified with a learned sequencing error correction model (DADA2 method),^79^ using the dada2 package in R. ASVs were assigned taxonomy using the GreenGenes database (version 13.8). ɑ-diversity was quantified as the number of observed ASVs, Shannon diversity, or phylogenetic diversity (PD) whole tree, on rarefied samples using the phyloseq package in R (version 3.4.0). Data were rarefied to 8,759 reads per sample (lowest 10% of reads, 352 samples retained out of 392 total) also using the phyloseq package in R. Rarefied data were only used for ɑ-diversity measures. β-diversity was calculated, and the analysis of variance using distance matrices was calculated with the adonis function (method = “bray”) in the vegan package in R (version 2.5.6).

### Circulating inflammatory markers

Cytokine data were generated from serum samples submitted to Olink Proteomics for analysis using their provided inflammation panel assay of 92 analytes (Olink INFLAMMATION). Significance was assessed using the siggenes package in R (SAM two-class paired between timepoint and unpaired between treatment arms and response group), FDR ≤ 0.05, q-value ≤ 0.1). Permutational Multivariate Analysis of Variance analysis was conducted using the vegan package in R (adonis, distance = “eu”).

### Untargeted serum metabolomics

Participant serum samples were analyzed for untargeted serum metabolomics and extracted in LC-MS grade methanol (4:1 v/v). Precipitation of proteins was conducted by incubating samples for 5 min at room temperature and centrifuging at 5,00×*g* for 10 min. Supernatant for each sample was then transferred, evaporated, and reconstituted in an internal standard mix (50% methanol). Metabolomics of each sample was analyzed on an LC-MS qTOF with reverse phase C19 positive, C18 negative, and HILIC positive methods as described previously.^80^ Annotation of compounds was completed using MSDIAL software^81^ and an authentic standard reference library. Quantification of metabolite levels was done using area under the curve for each annotated metabolite and normalized using the sum of internal standards for each individual sample.

### Statistical analysis of primary outcome

The primary outcome as listed on ClinicalTrials.gov was change in parameters defining metabolic syndrome (waist circumference, blood pressure, triglycerides, HDL-cholesterol, and fasting glucose) from baseline (week 0) to end of intervention (week 10) in participants with three out of the five parameters defined by the International Diabetes Foundation. Significant changes were evaluated by filtering the participants that fit these criteria (*n* = 21 for probiotic treatment arm, *n* = 13 for placebo treatment arm), followed by a paired *t*-test (within participants) and unpaired *t*-test (between probiotic vs. placebo treatment arms).

### Recursive feature random forest

To determine which metabolites were most predictive of response to the probiotic supplement (responder or non-responder), a recursive feature random forest (caret, rfeControl, number = 100, leave one out cross validation) was used. Data input was the participant-specific differences from end of intervention (week 10) to baseline (week −3). If a participant did not have both the baseline and end of intervention time point for a given experimental platform they were removed from analysis. All metabolite difference values were centered and scaled. The recursive feature random forest models returned the minimum feature set needed for highest accuracy.

### Multiple testing using significance analysis of microarrays (SAM)

The identification of parameters differentially expressed between treatment arms (unpaired) or within the same participant at different time points (paired) and estimation of the false discovery rate (FDR) was calculated using the siggenes package in R. To decrease redundant parameters of large feature sets, unsupervised parameter filtration was used. Circulating inflammatory markers and untargeted serum metabolomics were filtered to top 75% most varying parameters. Micro and macro nutritional intake were filtered to parameters non-zero in at least 50% of all food logs (95 parameters from 109). Intake for each participant was the average of total food logged during week 0 and week 10 of study. Significance for the SAM was defined as FDR ≤ 0.05 and a q-value ≤ 0.10.

### Permutational multivariate analysis of variance using distance matrices

To determine whether circulating serum inflammatory markers, untargeted serum metabolomics profiles, or ASV profiles were significantly different between the probiotic vs. placebo treatment arms or probiotic responders vs. probiotic non-responders and placebo treatment arms, analysis of variance using distance matrices was used. Input for both serum derived data types (inflammatory markers and metabolomics) was the difference from baseline (week −3) to end of intervention (week 10) into a Euclidean distance matrix. Input for the ASV profiles was the relative abundance of ASVs found in >25% of samples at the end of intervention (week 10) into a Bray–Curtis distance matrix. The adonis function (vegan package, R, permutations = 99) was used to test if there was a significant difference between probiotic vs. placebo treatment arms or probiotic responders vs. non-responders and placebo participants.

### Modeling ASV changes in relative abundance and presence/absence over time using a zero-inflated beta random effect model (ZIBR)

To identify differences in abundance and/or presence of taxa between the probiotic responders and non-responders, the zero-inflated beta regression model was fit using the ZIBR package in R. A filtered dataset was curated as described.^82^ ASVs were preprocessed using tip_glom (phyloseq package in R, h = 0.1), removed if they were non-characterized in GreenGenes, and filtered to only ASVs present in at least 25% of samples. Missing samples were filled with the average ASV abundance for each group at each timepoint. Taxa with significant baseline coefficients were filtered out to focus on the significant differences induced by the intervention.

## Supplementary Material

Supplemental Material

## Data Availability

Datasets and code for analysis are available at https://github.com/SonnenburgLab/project-probiotic-study/. Raw data files for 16S and metagenomic sequencing available at BioProject database
