# Supplementary material for: Randomized controlled trial demonstrates response to a probiotic intervention for metabolic syndrome that may correspond to diet
Source: Gut Microbes. 2023 Feb 19;15(1):2178794. doi: 10.1080/19490976.2023.2178794 (PMC9980610; doi:10.1080/19490976.2023.2178794)
Supplement: Supplemental Material [file KGMI_A_2178794_SM2466.zip › newTableS1_demographics.docx]

## Table S1, Related to Figure 1. Demographics table at screening.

|  | Probiotic (n=26) | Placebo (n=13) |
| --- | --- | --- |
| **Demographics (% of diet arm)** | | |
| Female | 14 (54%) | 8 (62%) |
| Male | 12 (46%) | 5 (38%) |
| Non-Hispanic | 23 (88%) | 13 (100%) |
| Hispanic | 3 (12%) | 0 (0%) |
| Asian | 4 (15%) | 2 (15%) |
| White | 17 (65%) | 9 (69%) |
| Black/African American | 2 (8%) | 2 (15%) |
| Married/Partnered | 18 (69%) | 8 (62%) |
| Divorced | 2 (8%) | 3 (23%) |
| Separated | 1 (4%) | 0 (0%) |
| Single/Never married | 5 (19%) | 2 (15%) |
| Some college | 1 (4%) | 3 (23%) |
| College graduate | 4 (15%) | 4 (31%) |
| Some post-graduate school | 3 (12%) | 0 (0%) |
| Post-graduate degree | 18 (69%) | 6 (46%) |
| Working full-time | 14 (54%) | 9 (69%) |
| Working part-time | 4 (15%) | 2 (15%) |
| Unemployed | 2 (8%) | 1 (8%) |
| Retired | 6 (23%) | 1 (8%) |
| Smokers | 0 (0%) | 0 (0%) |
| Non-smokers | 26 (100%) | 13 (100%) |
| **Anthropometrics (average+SD)** | | |
| Age (years) | 57 + 13 | 55 + 12 |
| WAIST (cm) | 102.8 + 10.4 | 102.6 + 10.0 |
| Weight (kg) | 87.2 + 17.1 | 87.1 + 15.1 |
| BMI | 30.2 + 4.2 | 30.3 + 4.7 |
| Systolic BP | 131 + 11 | 131 + 12 |
| Diastolic BP | 79 + 11 | 76 + 10 |
| **Blood Values (average+SD)** | | |
| Glucose (mg/dL) | 103 + 14 | 112 + 18 |
| Triglycerides (mg/dL) | 126 + 64 | 128 + 59 |
| HDL Cholesterol (mg/dL) | 50 + 11 | 50 + 15 |
