# Supplementary material for: Randomized controlled trial demonstrates response to a probiotic intervention for metabolic syndrome that may correspond to diet
Source: Gut Microbes. 2023 Feb 19;15(1):2178794. doi: 10.1080/19490976.2023.2178794 (PMC9980610; doi:10.1080/19490976.2023.2178794)
Supplement: Supplemental Material [file KGMI_A_2178794_SM2466.zip › newTableS3_primaryoutcome.docx]

## Table S3, Related to Figure 2. Primary outcome of change in metabolic syndrome parameters with 95% confidence intervals in participants with metabolic syndrome from beginning (week 0) to end (week 10) of intervention. Note: only includes participants with 3 parameters for metabolic syndrome or more.

|  | Probiotic (n=21) | Placebo (n=13) |
| --- | --- | --- |
| **Anthropometrics (average+SD)** | | |
| WAIST (cm) | 104.8 + 11.0 | 105.6 + 10.0 |
| Systolic BP | 128 + 13 | 129 + 14 |
| Diastolic BP | 78 + 9 | 78 + 9 |
| **Blood Values (average+SD)** | | |
| Glucose (mg/dL) | 98 + 14 | 104 + 25 |
| Triglycerides (mg/dL) | 139 + 68 | 132 + 50 |
| HDL Cholesterol (mg/dL) | 51 + 12 | 51 + 14 |
