# Supplementary material for: Randomized controlled trial demonstrates response to a probiotic intervention for metabolic syndrome that may correspond to diet
Source: Gut Microbes. 2023 Feb 19;15(1):2178794. doi: 10.1080/19490976.2023.2178794 (PMC9980610; doi:10.1080/19490976.2023.2178794)
Supplement: Supplemental Material [file KGMI_A_2178794_SM2466.zip › newTableS4_pvalues_relFig2A.docx]

## Table S4, Related to Figure 2. Adjusted p-values for primary outcome paired analysis of placebo and probiotic arms.

| **P-value from paired t-test p-value baseline (week -4) vs. intervention (week 10).** | | |
| --- | --- | --- |
| **Parameter** | **Probiotic (n=21)** | **Placebo (n=13)** |
| Triglycerides | 0.60 | 0.88 |
| Insulin | 0.86 | 0.99 |
| Glucose | 0.60 | 0.88 |
| HDL Cholesterol | 0.60 | 0.99 |
| Waist Circumference | 0.75 | 0.88 |
| Diastolic Blood Pressure | 0.60 | 0.88 |
| Systolic Blood Pressure | 0.60 | 0.88 |
| LDL Cholesterol | 0.60 | 0.99 |
| Alanine Transaminase | 0.75 | 0.50 |

Note: only includes participants with 3 parameters for metabolic syndrome or more
