# Supplementary material for: Randomized controlled trial demonstrates response to a probiotic intervention for metabolic syndrome that may correspond to diet
Source: Gut Microbes. 2023 Feb 19;15(1):2178794. doi: 10.1080/19490976.2023.2178794 (PMC9980610; doi:10.1080/19490976.2023.2178794)
Supplement: Supplemental Material [file KGMI_A_2178794_SM2466.zip › newTableS5_paired_pvalues_relFig2C.docx]

## Table S5, Related to Figure 2C. Adjusted p-values for paired t-test on percent change from baseline (avg. week -4, -2, 0) to end of intervention (avg. week 8, 10). Note: all participants included (responders n=14; non-responders n=12; placebo n=13).

| **Parameter** | **Probiotic Responders** | **Probiotic Non-responders** | **Placebo** |
| --- | --- | --- | --- |
| Triglycerides | **0.04** | 0.28 | 0.58 |
| Insulin | 0.14 | **0.03** | 0.63 |
| Glucose | 0.98 | **0.03** | 0.63 |
| HDL Cholesterol | 0.27 | 0.91 | 0.58 |
| Waist Circumference | 0.18 | 0.91 | 0.89 |
| Diastolic Blood Pressure | **0.04** | 0.91 | 0.98 |
| Systolic Blood Pressure | 0.07 | 0.91 | 0.89 |
| LDL Cholesterol | 0.98 | 0.91 | 0.89 |
| Alanine Transaminase | 0.13 | 0.91 | 0.58 |
