# Supplementary material for: Randomized controlled trial demonstrates response to a probiotic intervention for metabolic syndrome that may correspond to diet
Source: Gut Microbes. 2023 Feb 19;15(1):2178794. doi: 10.1080/19490976.2023.2178794 (PMC9980610; doi:10.1080/19490976.2023.2178794)
Supplement: Supplemental Material [file KGMI_A_2178794_SM2466.zip › newTableS6_demographics_RNR.docx]

## Table S6, Related to Figure 1. Demographics table at screening, probiotic responders and non-responders.

|  | Probiotic Responders (n=14) | Probiotic Non-responders (n=12) |
| --- | --- | --- |
| **Demographics (% of diet arm)** | | |
| Female | 7 (50%) | 7 (58%) |
| Male | 7 (40%) | 5 (42%) |
| Non-Hispanic | 11 (79%) | 12 (100%) |
| Hispanic | 3 (21%) | 0 (0%) |
| Asian | 1 (7%) | 3 (25%) |
| White | 8 (57%) | 9 (75%) |
| Black/African American | 2 (14%) | 0 (0%) |
| Married/Partnered | 9 (64%) | 9 (75%) |
| Divorced | 2 (14%) | 0 (0%) |
| Separated | 0 (0%) | 1 (8%) |
| Single/Never married | 3 (31%) | 2 (17%) |
| Some college | 1 (7%) | 0 (0%) |
| College graduate | 2 (14%) | 2 (17%) |
| Some post-graduate school | 2 (14%) | 1 (8%) |
| Post-graduate degree | 9 (64%) | 9 (75%) |
| Working full-time | 8 (57%) | 6 (50%) |
| Working part-time | 1 (7%) | 3 (25%) |
| Unemployed | 1 (7%) | 1 (8%) |
| Retired | 4 (29%) | 2 (17%) |
| Smokers | 0 (0%) | 0 (0%) |
| Non-smokers | 14 (100%) | 12 (100%) |
| **Anthropometrics (average+SD)** | | |
| Age (years) | 56 + 13 | 58 + 14 |
| WAIST (cm) | 102.2 + 10.9 | 103.5 + 10.3 |
| Weight (kg) | 88.8 + 19.3 | 85.5 + 14.9 |
| BMI | 30.6 + 4.9 | 29.7 + 3.3 |
| Systolic BP | 132 + 13 | 130 + 10 |
| Diastolic BP | 78 + 10 | 81 + 12 |
| **Blood Values (average+SD)** | | |
| Glucose (mg/dL) | 103 + 10 | 102 + 18 |
| Triglycerides (mg/dL) | 135 + 78 | 115 + 44 |
| HDL Cholesterol (mg/dL) | 50 + 11 | 49 + 13 |
