# Supplementary material for: Randomized controlled trial demonstrates response to a probiotic intervention for metabolic syndrome that may correspond to diet
Source: Gut Microbes. 2023 Feb 19;15(1):2178794. doi: 10.1080/19490976.2023.2178794 (PMC9980610; doi:10.1080/19490976.2023.2178794)
Supplement: Supplemental Material [file KGMI_A_2178794_SM2466.zip › newTableS8_unpaired_pvalues_relFig2C.docx]

## Table S8, Related to Figure 2C. Adjusted p-values for unpaired t-test on percent change from baseline (avg. week -4, -2, 0) to end of intervention (avg. week 8, 10). Note: all participants included (responders n=14; non-responders n=12; placebo n=13).

| **Parameter** | **Probiotic vs. Placebo** | **Probiotic Responders vs. Placebo** | **Probiotic Non-responders vs. Placebo** |
| --- | --- | --- | --- |
| Triglycerides | 0.76 | **0.021** | 0.76 |
| Insulin | 0.90 | 0.21 | 0.68 |
| Glucose | 0.21 | 0.63 | **0.045** |
| HDL Cholesterol | 0.76 | 0.27 | 0.97 |
| Waist Circumference | 0.90 | 0.47 | 0.97 |
| Diastolic Blood Pressure | 0.76 | 0.27 | 0.97 |
| Systolic Blood Pressure | 0.76 | 0.27 | 0.97 |
| LDL Cholesterol | 0.92 | 0.99 | 0.97 |
| Alanine Transaminase | 0.90 | 0.47 | 0.68 |
